# Huge Right Retroperitoneal Paraganglioma With a High Risk of Surgical Excision Treated Conservatively for 14 Years

**DOI:** 10.7759/cureus.21072

**Published:** 2022-01-10

**Authors:** Rania Naguib

**Affiliations:** 1 Department of Clinical Science, College of Medicine, Princess Nourah Bint Abdulrahman University, Riyadh, SAU

**Keywords:** paraganglioma, surgery, treatment, non‑functional paraganglioma, retroperitoneal paraganglioma

## Abstract

Paraganglioma is an uncommon type of neuroendocrine tumor capable of secreting neuropeptides and catecholamines. Retroperitoneal paragangliomas are rare neoplasms that originate from chromaffin cells that secrete catecholamines in the sympathetic ganglia. Tumor reduction and management of excessive catecholamine secretion are the key treatment goals. Surgery is the choice of treatment modality when tumors are amenable to resection because of their malignant potential. Currently, high-dose metaiodobenzylguanidine (MIBG) radiation and chemotherapy are adjuvant therapy to surgery. This case is reported to demonstrate a good prognosis in a conservatively managed, huge, non-functional retroperitoneal paraganglioma for 14 years. This provides alternative options to the traditional surgical treatment in patients who refuse, are unfit for surgery, or have complex surgery, which carries a high mortality rate with analysis of follow-up modalities. Meanwhile, a review of the relevant literature was conducted in order to figure out the prognosis.

## Introduction

Pheochromocytomas and paragangliomas are neuroendocrine tumors that develop from chromaffin-containing tissue. These tumors have a total prevalence of about 1:2500 and 1:6500, respectively [1]. Paragangliomas can be benign or malignant, with a malignant paraganglioma rate of 93 per 400 million people. Paragangliomas are primarily sporadic. However, they can be linked to syndromes such as multiple endocrine neoplasia type 2, von Hippel-Lindau disease, and neurofibromatosis type 1 [2]. Paragangliomas arise from the sympathetic chain in the chest, abdomen, and pelvis. On the other hand, pheochromocytomas originate from identical cells in the adrenal glands [1].

Some tumors (termed functional paragangliomas) have been found to synthesize, store, and secrete catecholamines. This results in elevated levels of urine/serum catecholamines and typical clinical symptoms such as episodic headache (72%), sweating (69%), and palpitations (51%). Approximately 10-15% of such tumors are non-functional. These tumors do not secret catecholamines and do not present with catecholamine hypersecretion manifestations. Instead, they are frequently space-occupying lesions originating from parasympathetic nerves, which might produce pain due to a mass impact on nearby structures. In addition, they are frequently locally invasive and have a high rate of local recurrence [2, 3]. This case report aims to analyze the treatment options, follow-up modalities, and prognostic outcomes of a non-functional, huge retroperitoneal paraganglioma followed for 14 years.

## Case presentation

**Figure 1:**
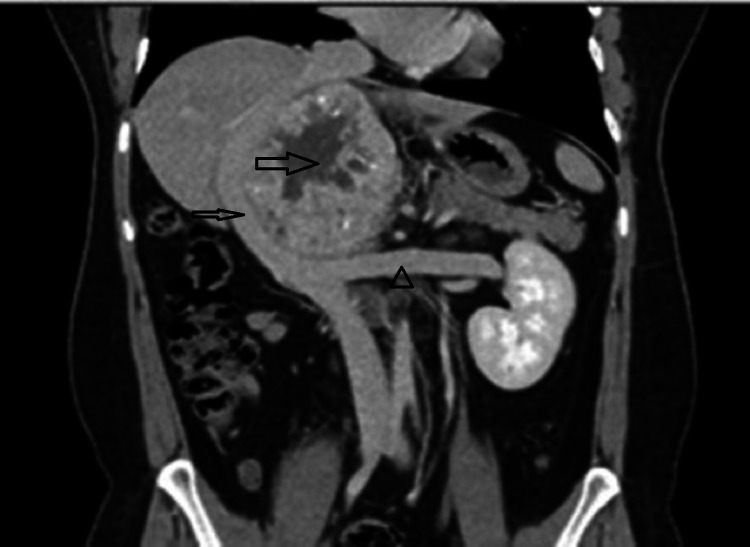
CT scan (coronal view) showing huge right retroperitoneal tumor with central necrosis (thick arrow) pushing the IVC (thin arrow) anteriorly to the right. The left renal vein (arrowhead) is being pushed inferiorly. The IVC and left renal vein are being stretched over the tumor without signs of an invasion. IVC: Inferior vena cava.

A 29-year-old male patient was referred to the surgical oncology department from his local hospital as a case of huge, non-functional abdominal paraganglioma in 2008. His main complaints were mild, tolerable heartburn and epigastric pain. A confirmatory CT scan showed a huge right retroperitoneal tumor with mass effect on the stomach and other organs, with a critical position posterior to the inferior vena cava (IVC) and pushing it forward. It also has critical relations to the renal veins (Figure 1).

**Figure 2:**
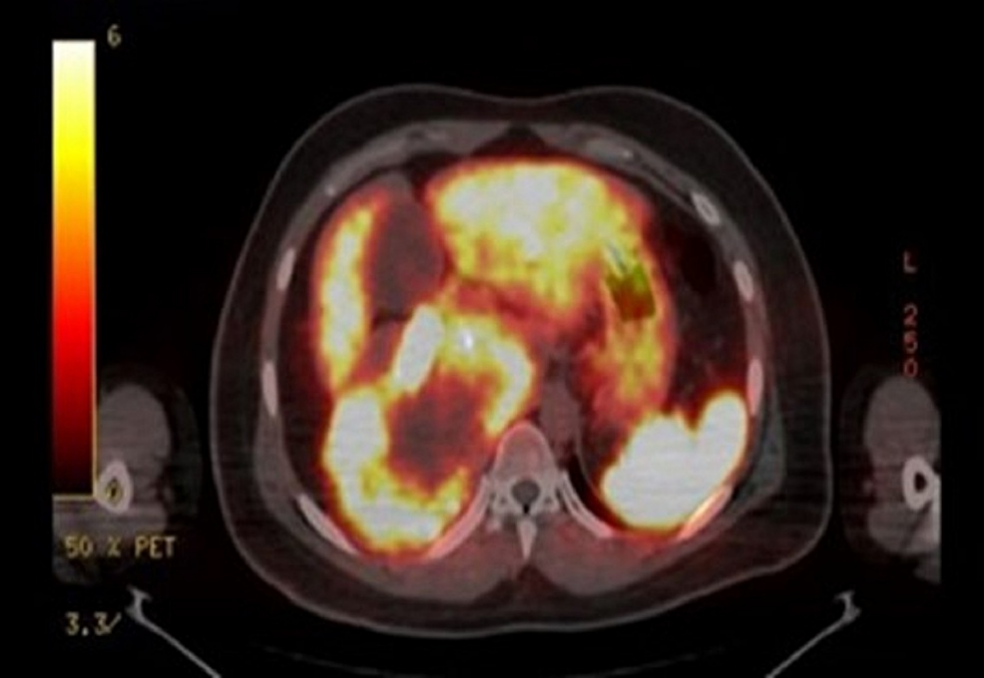
PET-CT scan showing radiogallium heterogeneously avid, right retroperitoneal hypervascular mass, with central necrosis and multiple foci of cystic changes. There is gross interval stability in the mass size and persistent non-visualization of the right kidney. PET: Positron emission tomography.

**Figure 3:**
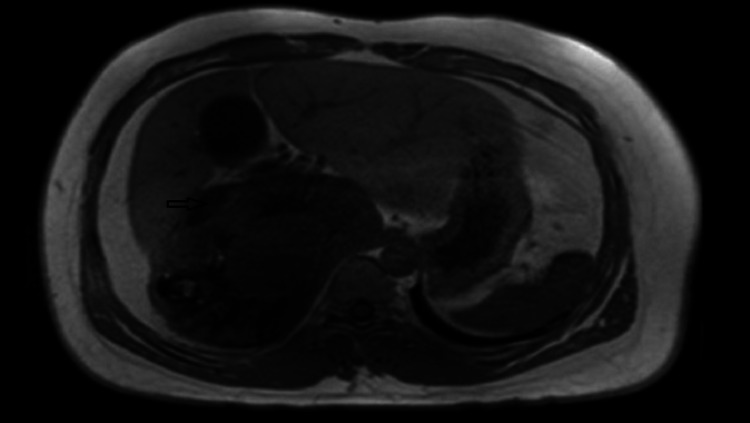
MRI showing a huge right retroperitoneal tumor pushing the IVC (arrow) without invasion. IVC: Inferior vena cava.

The diagnosis of retroperitoneal paraganglioma was proved by fine needle aspiration (FNA). The pathology report showed that the tumor cells revealed positive staining for chromogranin (a neuroendocrine marker) and negative for Melan-A (melanocytic differentiation marker). The surgical oncology team discussed the high risk of tumor resection with the patient and his family. They were informed about the huge size of the tumor and its critical position concerning the major blood vessels like the IVC, renal veins, and other abdominal organs. The patient refused to take the risk of such an operation, especially since he had only mild complaints that did not affect his life. The decision was that the patient is better managed conservatively, so he was referred to the endocrine department. The patient received chemotherapy followed by radiotherapy in 2009 with no response. In a trial to shrink the tumor and control its size, embolization was done twice during 2010 with no significant effect. He also received five cycles of metaiodobenzylguanidine (MIBG) therapy from 2010 to 2013 with a total dose of 480 millicuries with an insignificant response. The patient is currently receiving octreotide. Follow-up by CT scan was done twice yearly. It demonstrated stable right side retroperitoneal paraganglioma with a similar mass effect and no definite IVC invasion. Positron emission tomography (PET) gallium CT scan (Figure 2) and MRI (Figure 3) were done alternatively biannually for better soft tissue delineation and to exclude IVC invasion. All showed stability of the tumor over the years with no progression. Over these years, no evidence of metastasis was detected. So, for now, he is on regular follow-up in the endocrine clinic with imaging every six months. 

## Discussion

Retroperitoneal paragangliomas are extremely rare tumors. Non-functional retroperitoneal forms are even more infrequent and are usually isolated [3]. The exact incidence of retroperitoneal paragangliomas is unknown, but males are more commonly affected than females. The majority of patients are diagnosed between the ages of 30 and 45 [4]. Clinical manifestations of paragangliomas vary depending on tumor's location and size, catecholamine secretory function, and extent of spread [1]. Non-functional retroperitoneal paragangliomas are distinguished by their asymptomatic profile and normal catecholamine levels in the urine and blood [3]. Patients with retroperitoneal paraganglioma frequently present with abdominal pain or a palpable mass [4].

Paragangliomas are usually benign. However, they can be locally invasive. They can also turn malignant with distant metastasis. Because of the possibility of malignant transformation of paragangliomas, and since paragangliomas do not respond well to chemotherapy or radiation, surgical excision is still the preferred treatment option [1]. 

Surgical debulking has been shown to improve overall survival, possibly by limiting the possibility of metastatic spread. Surgery allows for a significant improvement in prognosis, with a five-year and ten-year survival rate without relapse of 75% and 45%, respectively. In the case of metastatic forms, the median overall survival is approximately three years, and in the case of incomplete resection, the median overall survival is approximately four years. In metastatic forms, complementary therapies such as chemotherapy or radiation therapy produce a positive response in approximately half of the cases, but have no significant influence on prognosis [5]. Close postoperative monitoring with CT/MRI and PET scans may also be used to monitor for malignant potential or progression. This should begin three months after surgery and continue biannually for the first three years. Lifelong follow-up is advised [4, 6]. Radical resection is possible in only 75% of cases [5].

Some paragangliomas represent a challenge in management due to high vascularity [7]. Another challenge is that they sometimes attain huge sizes. A third challenge is that they arise from very critical locations near vital organs or major blood vessels. These challenges can cause surgical resection to carry a very high risk of complications or even death [8]. In the case of surgically unresectable tumors, it is recommended to reduce the tumor's size by using chemotherapy, radiation therapy, or embolization. Octreotide is another option in treating inoperable paragangliomas [8].

The patient’s family refused surgery in the current case after learning about the possible operative risks. The patient is on a regular follow-up using PET-CT scan and MRI for the delineation of the outline of the tumor, detection of a change in size, determination of its relationship with the surrounding organs, and early detection of possible progression. The patient tumor size has been stable for 14 years with no deleterious effects, and he is conducting a normal life with no effect on his daily activities. As surgical techniques and systemic therapies advance, randomized clinical trials will be crucial in determining the best treatment plan for these patients.

## Conclusions

Non‑functional retroperitoneal paragangliomas are a rare group of tumors. Surgical excision is the best treatment of choice. Adjuvant radiotherapy or chemotherapy has a minimal role. Regular follow-up with a CT scan and/or MRI is necessary to detect tumor progression. When confronted with a difficult huge, non-functional retroperitoneal paraganglioma, with a high risk of surgical intervention and refusing patient, conservative treatment with follow-up is an option with a good prognosis.
